# Polyserine domains are toxic and exacerbate tau pathology in mice

**DOI:** 10.1073/pnas.2527425122

**Published:** 2026-01-02

**Authors:** Meaghan Van Alstyne, Vanessa L. Nguyen, Charles A. Hoeffer, Roy Parker

**Affiliations:** ^a^Department of Biochemistry, University of Colorado, Boulder, CO 80303; ^b^HHMI, University of Colorado, Boulder, CO 80303; ^c^Department of Integrative Physiology, University of Colorado, Boulder, CO 80303; ^d^BioFrontiers Institute, University of Colorado, Boulder, CO 80303

**Keywords:** tau, polyserine, neurodegeneration, tauopathy, repeat expansion

## Abstract

Polyserine domains mediate the association of some nuclear RNA-binding proteins with cytoplasmic tau aggregates occurring in tauopathy models and patient samples. In cell lines, polyserine domains mediate colocalization with tau aggregates and promote formation, suggesting that the cytoplasmic mislocalization of polyserine-containing proteins could contribute to human disease. Moreover, polyserine can be produced by repeat-associated non-AUG translation in CAG repeat expansion diseases. However, whether polyserine expressed in a mammalian brain is toxic and/or can exacerbate tau pathology is unknown. We used AAV9-mediated delivery to express a 42-repeat polyserine protein in wild-type and tau transgenic mouse models. We observe that polyserine expression has toxic effects in wild-type animals indicated by reduced weight, behavioral abnormalities, and a striking loss of Purkinje cells. Moreover, in the presence of a pathogenic variant of human tau, polyserine exacerbates disease markers such as phosphorylated and insoluble tau levels and the seeding capacity of brain extracts. These findings demonstrate that polyserine domains can promote tau-mediated pathology in a mouse model and support the hypothesis that polyserine-containing proteins could contribute to the progression of human tauopathies.

## Significance

This study evaluates the toxic effects of polyserine domains in vivo. Polyserine and endogenous polyserine-containing proteins have been shown to enrich in tau aggregates across models and in human postmortem tissues. Further, polyserine proteins are expressed in the context of CAG repeat expansion diseases such as spinocerebellar ataxia. Through viral-mediated delivery, this study demonstrates that expression of polyserine-containing proteins leads to toxicities in both wild-type and tau transgenic mouse models. Toxic effects independent of genotype include motor impairment and the loss of Purkinje cells in the cerebellum. Further, in mice expressing human mutant tau, polyserine expression exacerbates markers of tau pathology. These results support further investigation of polyserine in the pathogenesis of tauopathies and CAG repeat expansion disorders.

The microtubule-binding protein tau is a key player in several neurodegenerative diseases termed tauopathies (1). In disease, tau transitions into an insoluble state, forming intracellular aggregates—a hallmark feature of pathology (1). Multiple factors can contribute to tau pathobiology including posttranslational modifications or disease-associated mutations that promote transition from a microtubule-bound state to an aggregated fibrillar form characteristic of disease (2). Moreover, in vitro polyanionic cofactors such as RNA accelerate formation of fibrillar aggregates (3, 4). However, how potential cellular cofactors affect tau fibrillization in mammalian neurons remains incompletely understood.

We and others have demonstrated that the RNA-binding proteins SRRM2 and Pinin mislocalize to tau aggregates in cell models, animal models and postmortem patient tissue (5, 6). Long stretches of serine repeats or serine-rich regions drive this association with tau (7). Interestingly, in cellular models, polyserine (polySer)-containing proteins or exogenous polySer form assemblies that are preferred sites of tau aggregation (7). Further, the levels of polySer-containing proteins correlate with the extent of tau aggregation (7). However, whether polySer modulates tau aggregation and pathology in animal models is unknown.

In addition to being contained within proteins, polySer proteins can be endogenously expressed in the context of repeat expansion diseases. Specifically, in spinocerebellar ataxia 8 (SCA8) polySer-containing proteins are expressed through repeat-associated non-AUG (RAN) translation of transcripts derived from a CAG expansion translating in the AGC frame (8). In this context, polySer has been shown to accumulate in white matter regions where it coincides with demyelination and degeneration (8). Moreover, polySer proteins are also expressed from the CAG expansion causal in Huntington’s disease (HD) and are present in striatal regions prominently affected in disease (9, 10). Thus, polySer proteins are expressed across repeat expansion diseases where they may contribute to neurotoxicity. However, the toxic properties of polySer in the mammalian brain remain incompletely understood.

Here, we expressed polySer in wild-type and tau transgenic mouse models to determine the effects of polySer expression alone as well as potential modulation of tau pathology. We observe that polySer induces behavioral abnormalities in wild-type mice that coincide with a striking loss of Purkinje cells and gliosis. In addition, polySer exacerbates disease-relevant markers in tau transgenic mice. These observations provide evidence of polySer toxicity in vivo that support a potential role in the pathogenesis of CAG repeat expansion diseases. Further, they demonstrate that levels of polySer modulate tau pathology in an animal model, consistent with the hypothesis that increased association of polySer-containing proteins and tau could contribute to the progression of human tauopathies.

## Results

### AAV9-Mediated Expression of Polyserine Induces Toxicity in Wild-Type and Tau Transgenic Mice.

**Fig. 1.**
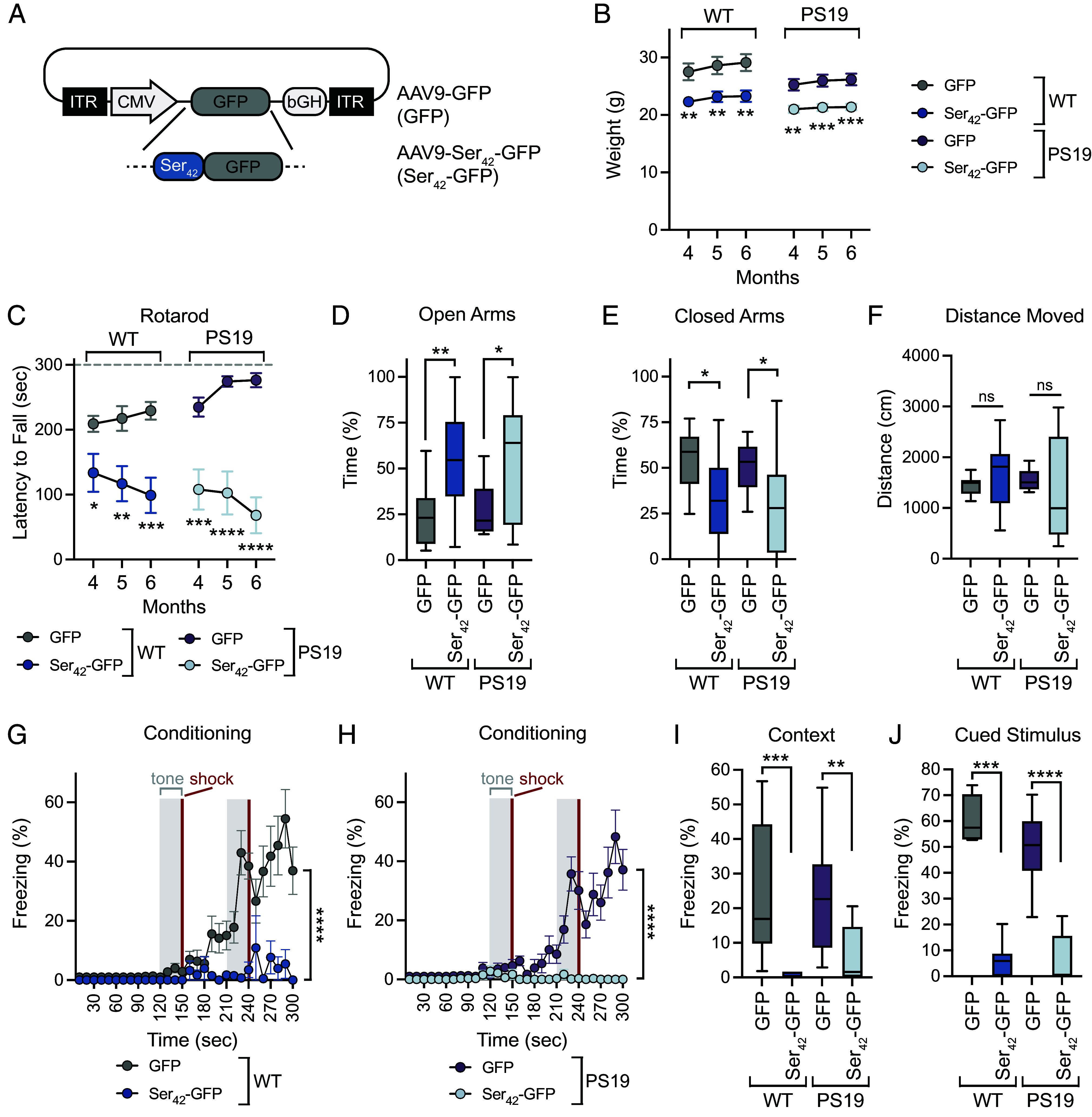
AAV9-mediated overexpression of polyserine leads to motor dysfunction in wild-type and tau transgenic mice. (*A*) Schematic of AAV constructs for in vivo expression of GFP and Ser_42_-GFP transgenes. (*B*) Weight of WT and PS19 tau transgenic animals injected with 1 × 10^11^ vgs of AAV9-GFP [WT n = 13 (6F, 7M); PS19 n = 14 (7F, 7M)] or AAV9-Ser_42_-GFP [WT n = 14 (7F, 7M); PS19 n = 13 (7F, 6M)] per animal by ICV injection at P1. Data represent mean and SEM. Statistics performed between GFP and Ser_42_-GFP-injected animals within paired genotypes with two-way ANOVA and Sidak’s multiple comparisons test. (*C*) Latency to fall on rotarod assay of AAV9-GFP [WT n = 13 (6F, 7M); PS19 n = 14 (7F, 7M)] or AAV9-Ser_42_-GFP [WT n = 14 (7F, 7M); PS19 n = 13 (7F, 6M)] WT or PS19 animals injected as in (*B*). Data represent mean and SEM. Statistics performed between GFP and Ser_42_-GFP-injected animals within paired genotypes with two-way ANOVA and Sidak’s multiple comparisons test. (*D*) Time spent on open arms in elevated plus maze assay at 6 mo for WT [GFP n = 14 (5F, 9M); Ser_42_-GFP n = 14 (7F, 7M)] and PS19 [GFP n = 14 (7F, 7M); Ser_42_-GFP n = 12 (7F, 5M)] animals injected with AAV9 as in (*B*). The box and whisker plot represents median, interquartile range, and error determined by Tukey’s method. Statistics performed with the Mann–Whitney test. (*E*) Time spent on closed arms in elevated plus maze assay at 6 mo for WT [GFP n = 14 (5F, 9M); Ser_42_-GFP n = 14 (7F, 7M)] and PS19 [GFP n = 14 (7F, 7M); Ser_42_-GFP n = 12 (7F, 5M)] animals injected with AAV9 as in (*B*). The box and whisker plot represents median, interquartile range, and error determined by Tukey’s method. Statistics performed with the Mann–Whitney test. (*F*) Distance moved in elevated plus maze assay at 6 mo for WT [GFP n = 14 (5F, 9M); Ser_42_-GFP n = 14 (7F, 7M)] and PS19 [GFP n = 14 (7F, 7M); Ser_42_-GFP n = 12 (7F, 5M)] animals injected with AAV9 as in (*B*). The box and whisker plot represents median, interquartile range, and error determined by Tukey’s method. Statistics performed with the Mann–Whitney test. (*G*) Percent freezing during conditioning period in fear conditioning assay at 6 mo for WT [GFP n = 11 (3F, 8M); Ser_42_-GFP n = 7 (4F, 3M)] animals injected with AAV9 as in (*B*). The box and whisker plot represents median, interquartile range, minimum and maximum. Statistics performed with two-way ANOVA and Sidak’s multiple comparisons test. (*H*) Percent freezing during conditioning period in fear conditioning assay at 6 mo for PS19 [GFP n = 14 (5F, 9M); Ser_42_-GFP n = 8 (5F, 3M)] animals injected with AAV9 as in (*B*). The box and whisker plot represents median, interquartile range, minimum and maximum. Statistics performed with two-way ANOVA and Sidak’s multiple comparisons test. (*I*) Percent freezing during contextual testing following conditioning in fear conditioning assay at 6 mo for WT [GFP n = 11 (3F, 8M); Ser_42_-GFP n = 7 (4F, 3M)] and PS19 [GFP n = 14 (5F, 9M); Ser_42_-GFP n = 8 (5F, 3M)] animals injected with AAV9 as in (*B*). The box and whisker plot represents median, interquartile range, and error determined by Tukey’s method. Statistics performed with the Mann–Whitney test. (*J*) Percent freezing during periods of cued stimulus (CS) following conditioning in fear conditioning assay at 6 mo for WT [GFP n = 11 (3F, 8M); Ser_42_-GFP n = 7 (4F, 3M)] and PS19 [GFP n = 14 (5F, 9M); Ser_42_-GFP n = 8 (5F, 3M)] animals injected with AAV9 as in (*B*). The box and whisker plot represents median, interquartile range, and error determined by Tukey’s method.

To investigate the effect of polySer domains in vivo, we utilized AAV9 gene delivery to express a GFP control or a GFP-tagged repeat of 42 serines (Ser_42_), the length of the longest polySer stretch in SRRM2 (5, 7). AAV9 was delivered to the central nervous system (CNS) by intracerebroventricular injection at P1 to both wild-type (WT) and tau mice harboring a 1N4R human tau transgene with a P301S disease-linked mutation (PS19) (Fig. 1*A*) (11).

A comparison of Ser_42_-GFP-injected mice with GFP controls showed polySer-induced toxicities in WT and PS19 animals. Notably, Ser_42_-GFP mice had reduced weight relative to GFP-injected animals of the same genotype (Fig. 1*B*). Furthermore, Ser_42_-GFP-injected mice display irregular motor behaviors such as gait abnormalities, head tilt, and circling phenotypes (Movies S1–S3). Reflecting these defects in locomotor function, Ser_42_-GFP-injected WT and PS19 mice had reduced performance on the rotarod assay from four to six months of age (Fig. 1*C*). While weight differences between groups were evident they do not explain locomotor differences as healthy lower-weight mice typically perform better on the rotarod assay (12).

Additional behavioral assays also highlight differences in Ser_42_-GFP-injected animals. In the elevated plus maze assay, Ser_42_-GFP mice spent increased time in open arms and reduced time in closed arms relative to GFP-injected WT or PS19 animals, while the total distance moved was not significantly different (Fig. 1 *D*–*F*). This suggests Ser_42_-GFP-injected mice have reduced natural aversion to exposed areas in this assay consistent with anxiolytic impacts of Ser_42_-GFP expression. In the open-field assay, GFP or Ser_42_-GFP-expressing mice had no significant differences in the time spent in the center (*SI Appendix*, Fig. S1 *A* and *B*). Still, WT Ser_42_-GFP-injected animals showed an increase in the total distance moved relative to controls which likely corresponds to mild hyperactivity in this setting (*SI Appendix*, Fig. S1*C*).

We also tested whether Ser_42_-GFP expression affected learning and associative memory in a fear conditioning assay. We observed that Ser_42_-GFP mice with or without a tau transgene show significantly reduced freezing during conditioning, indicating profound learning deficits (Fig. 1 *G* and *H*). As expected, reduced freezing is also evident during subsequent testing with contextual or cued stimuli (Fig. 1 *I* and *J*), as well as before cued stimuli (*SI Appendix*, Fig. S1*D*). These results are consistent with Ser_42_-GFP mice from either genotype failing to associate context or tone with shock thus exhibiting severe learning deficits.

These results collectively show that AAV9-mediated expression of polySer in the CNS leads to motor and behavioral dysfunction in both WT and tau transgenic animals.

### Polyserine Overexpression Induces Purkinje Cell Loss and Gliosis.

As Ser_42_-GFP expression induced locomotor deficiencies, we next looked for morphological changes that could underlie the observed phenotypes. Of particular interest were Purkinje cells due to their critical role in coordinating movement and the strong tropism of AAV9 for this cell type (13, 14).

**Fig. 2.**
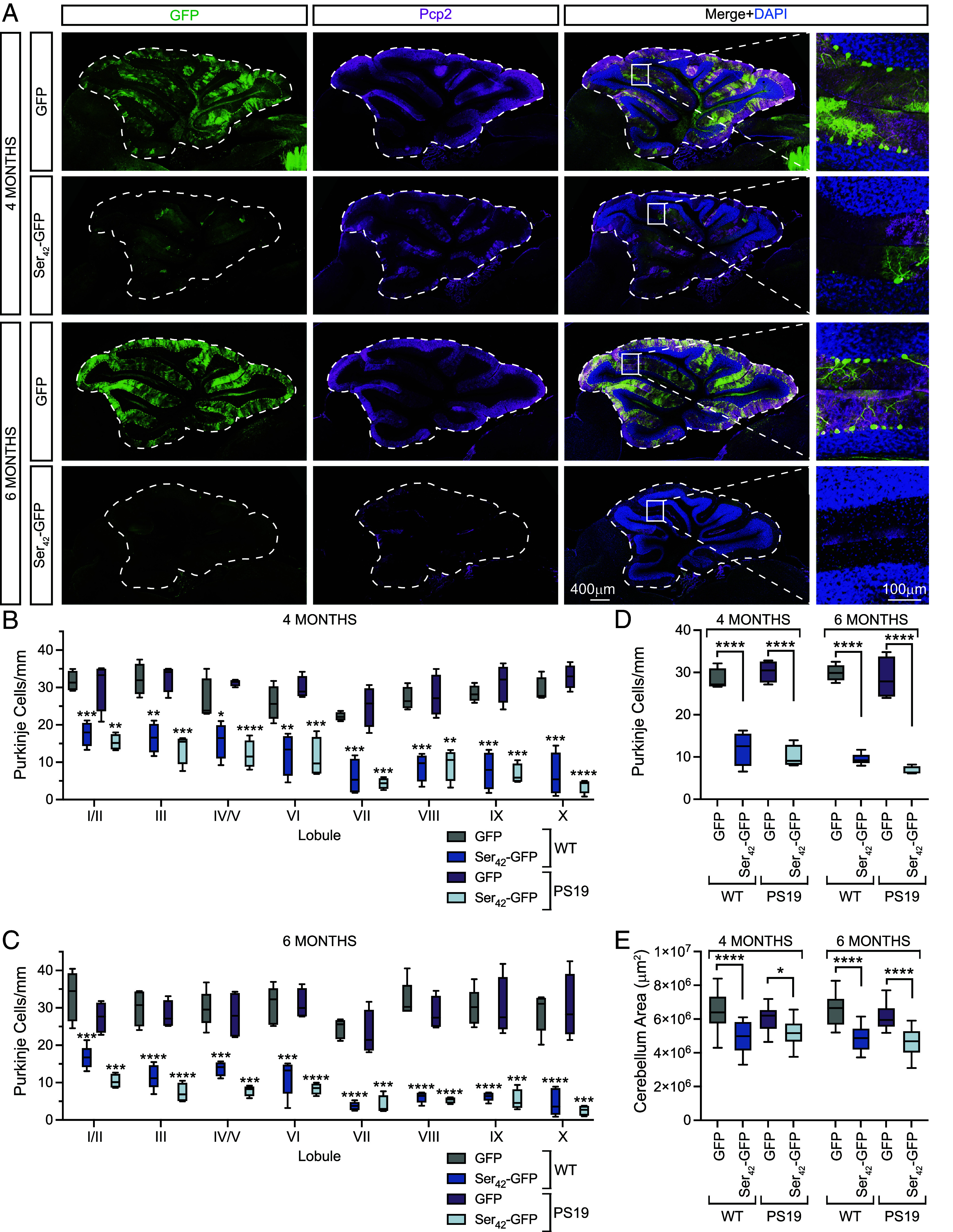
Polyserine expression induces Purkinje cell loss. (*A*) Immunostaining of DAPI (blue), GFP (green), and Pcp2 (magenta) in the cerebellum of WT animals at 4 and 6 mo of age injected with 1 × 10^11^ vgs of AAV9-GFP or AAV9-Ser_42_-GFP. Grey regions indicate areas outside of the acquired field of view, added for uniform panel formatting. (*B*) Quantification of Purkinje cell density of lobules from animals as in (*A*) at 4 mo of age. The box and whisker plot represents median, interquartile range, and error determined by Tukey’s method. n = 4 animals per group. WT [GFP (1F, 3M); Ser_42_-GFP (3F, 1M)], PS19 [GFP (2F, 2M); Ser_42_-GFP (2F, 2M)]. Statistics performed within genotypes for each lobule with the unpaired *t* test. (*C*) Quantification of Purkinje cell density of lobules from animals as in (*A*) at 6 mo of age. The box and whisker plot represents median, interquartile range, and error determined by Tukey’s method. WT [GFP n = 5 (2F, 3M); Ser_42_-GFP n = 5 (3F, 2M)], PS19 [GFP n = 4 (3F, 1M); Ser_42_-GFP n = 4 (2F, 2M)] Statistics performed within genotypes for each lobule with the unpaired *t* test. (*D*) Quantification of Purkinje cell density across all lobules as in (*A*) at 4 WT [GFP n = 4 (1F, 3M); Ser_42_-GFP n = 4 (3F, 1M)], PS19 n = 4 [GFP (2F, 2M); Ser_42_-GFP n = 4 (2F, 2M)] and 6 WT [GFP n = 5 (2F, 3M); Ser_42_-GFP n = 5 (3F, 2M)], PS19 [GFP n = 4 (3F, 1M); Ser_42_-GFP n = 4 (2F, 2M)]. Statistics performed for each timepoint with one-way ANOVA and Tukey’s multiple comparisons test. (*E*) Quantification of cross-sectional area of the cerebellum of WT and PS19 animals at 4 WT [GFP n = 6 (3F, 3M); Ser_42_-GFP n = 4 (3F, 1M)], PS19 [GFP n = 5 (3F, 2M); Ser_42_-GFP n = 5 (3F, 2M)] and 6 WT [GFP n = 6 (2F, 4M); Ser_42_-GFP n = 6 (3F, 3M), PS19 (GFP n = 6 (4F, 2M); Ser_42_-GFP n = 6 (3F, 3M)] months of age injected with AAV9 as in (*A*). The box and whisker plot represents median, interquartile range, and error determined by Tukey’s method. Data represent three sections per biological replicate. Statistics performed for each timepoint with one-way ANOVA and Tukey’s multiple comparisons test.

We performed immunostaining for GFP and Pcp2—a Purkinje cell marker—on cerebellum sections from GFP and Ser_42_-GFP-injected WT or PS19 transgenic animals. Consistent with previous reports, we observed high transduction of Purkinje cells in GFP-injected controls (Fig. 2*A* and *SI Appendix*, Fig. S2*A*) (14). Strikingly, Purkinje cells were dramatically lost across all lobules in Ser_42_-GFP-injected WT and PS19 mice relative to GFP-injected controls at four months of age (Fig. 2 *B* and *D*) which was further pronounced by six months (Fig. 2 *C* and *D*). We also measured the cross-sectional area of the cerebellum and observed a significant reduction in Ser_42_-GFP-injected animals relative to controls, consistent with the observed loss of Purkinje cells (Fig. 2*E*).

In contrast to Purkinje cells, hippocampal neurons—also highly transduced by AAV9—did not show notable loss in Ser_42_-GFP-injected animals (*SI Appendix*, Fig. S2*B*). This may be a consequence of Purkinje cells expressing higher levels of transgene and/or being particularly susceptible to polySer-induced toxicity. To gain insight into the mechanisms of Purkinje cell loss, we performed TUNEL staining to measure DNA fragmentation on cerebellar sections of GFP and Ser_42_-GFP-injected WT animals. While we observed staining in a DNAse-treated positive control, we did not observe any TUNEL positivity in GFP+ Purkinje cells, suggesting they are not lost through apoptosis (*SI Appendix*, Fig. S3).

**Fig. 3.**
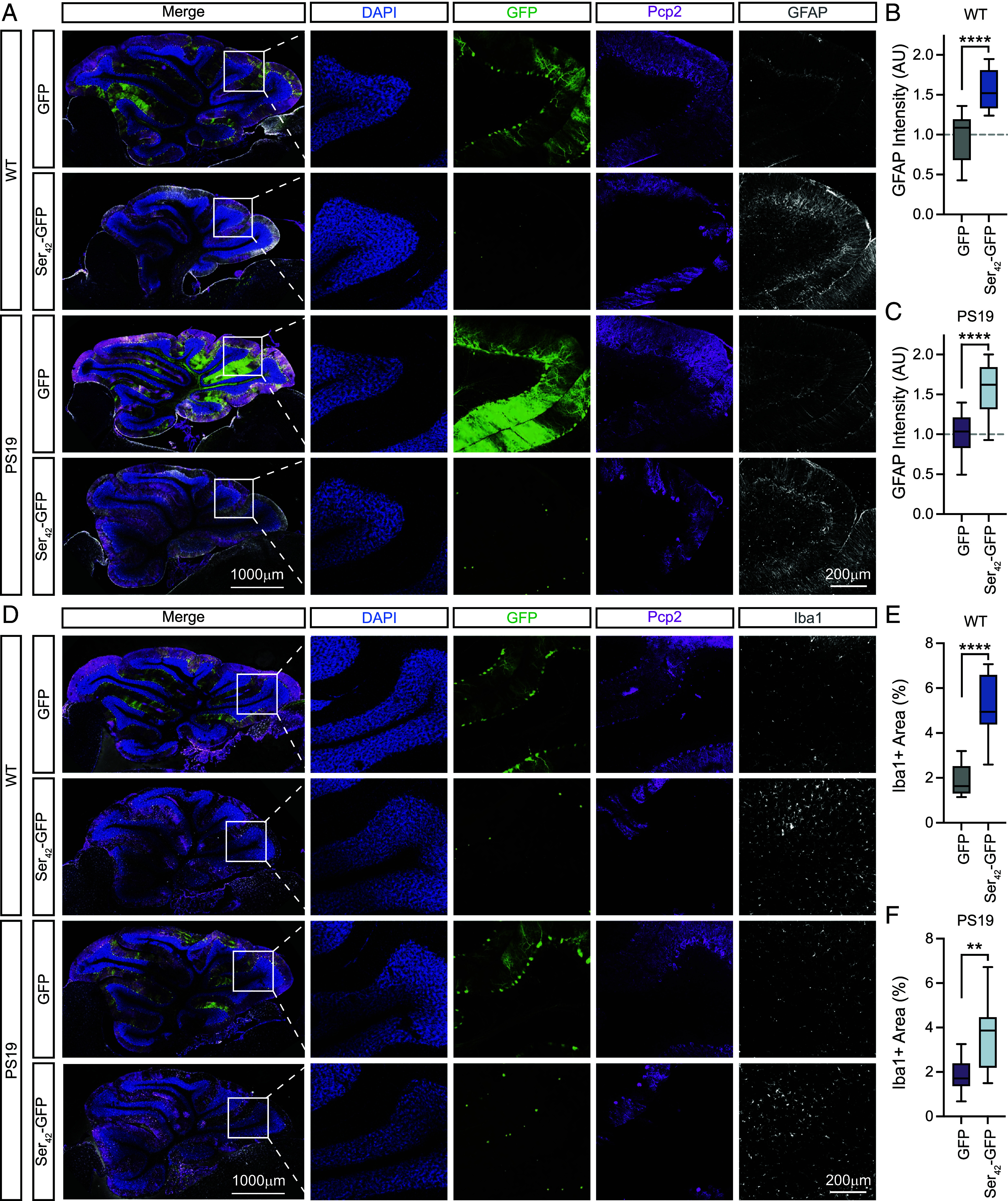
Polyserine expression in Purkinje cells induces gliosis. (*A*) Immunostaining of DAPI (blue), GFP (green), Pcp2 (magenta), and GFAP (gray) in the cerebellum of WT and PS19 animals at 4 mo of age injected with 1 × 10^11^ vgs of AAV9-GFP or AAV9-Ser_42_-GFP. Grey regions indicate areas outside of the acquired field of view, added for uniform panel formatting. (*B*) Quantification of GFAP intensity in the cerebellum for WT animals as in (A). The box and whisker plot represents median, interquartile range, and error determined by Tukey’s method. n = 4 animals per group. WT [GFP (1F, 3M); Ser_42_-GFP (3F, 1M)]. Statistics performed with the unpaired *t* test. (*C*) Quantification of GFAP intensity in the cerebellum for PS19 animals as in (A). The box and whisker plot represents median, interquartile range, and error determined by Tukey’s method. n = 4 animals per group. PS19 [GFP (2F, 2M); Ser_42_-GFP (2F, 2M)]. Statistics performed with the unpaired *t* test. (*D*) Immunostaining of DAPI (blue), GFP (green), Pcp2 (magenta), and Iba1 (gray) in the cerebellum of WT and PS19 animals at 4 mo of age injected with 1 × 10^11^ vgs of AAV9-GFP or AAV9-Ser_42_-GFP. Grey regions indicate areas outside of the acquired field of view, added for uniform panel formatting. (*E*) Quantification of Iba1-positive area in the cerebellum for WT animals as in (*A*). The box and whisker plot represents median, interquartile range, and error determined by Tukey’s method. n = 4 animals per group. WT [GFP (1F, 3M); Ser_42_-GFP (3F, 1M)]. Statistics performed with the unpaired *t* test. (*F*) Quantification of Iba1-positive area in the cerebellum for PS19 animals as in (*A*). The box and whisker plot represents median, interquartile range, and error determined by Tukey’s method. n = 4 animals per group. PS19 [GFP (2F, 2M); Ser_42_-GFP (2F, 2M)]. Statistics performed with the unpaired *t* test.

To assess whether Purkinje cell loss led to gliosis, we monitored astrocyte and microglia presence in the cerebellum. We performed immunostaining with the astrocyte marker GFAP at 4 mo of age (Fig. 3*A*) and observed a significant increase in GFAP intensity in Ser_42_-GFP-injected WT or PS19 animals relative to GFP controls—which was particularly notable in regions of pronounced Purkinje cell loss (Fig. 3 *B* and *C*). Next, we performed immunostaining for the microglial marker Iba1 (Fig. 3*D*). Quantification demonstrated increased Iba1 coverage in the cerebellum of Ser_42_-GFP-injected WT or PS19 animals relative to GFP controls (Fig. 3 *E* and *F*).

Thus, increased polySer domain levels lead to Purkinje cell loss and gliosis in the cerebellum, likely underlying some of the observed locomotor abnormalities.

### Polyserine Accumulates in Neuronal Nuclei.

**Fig. 4.**
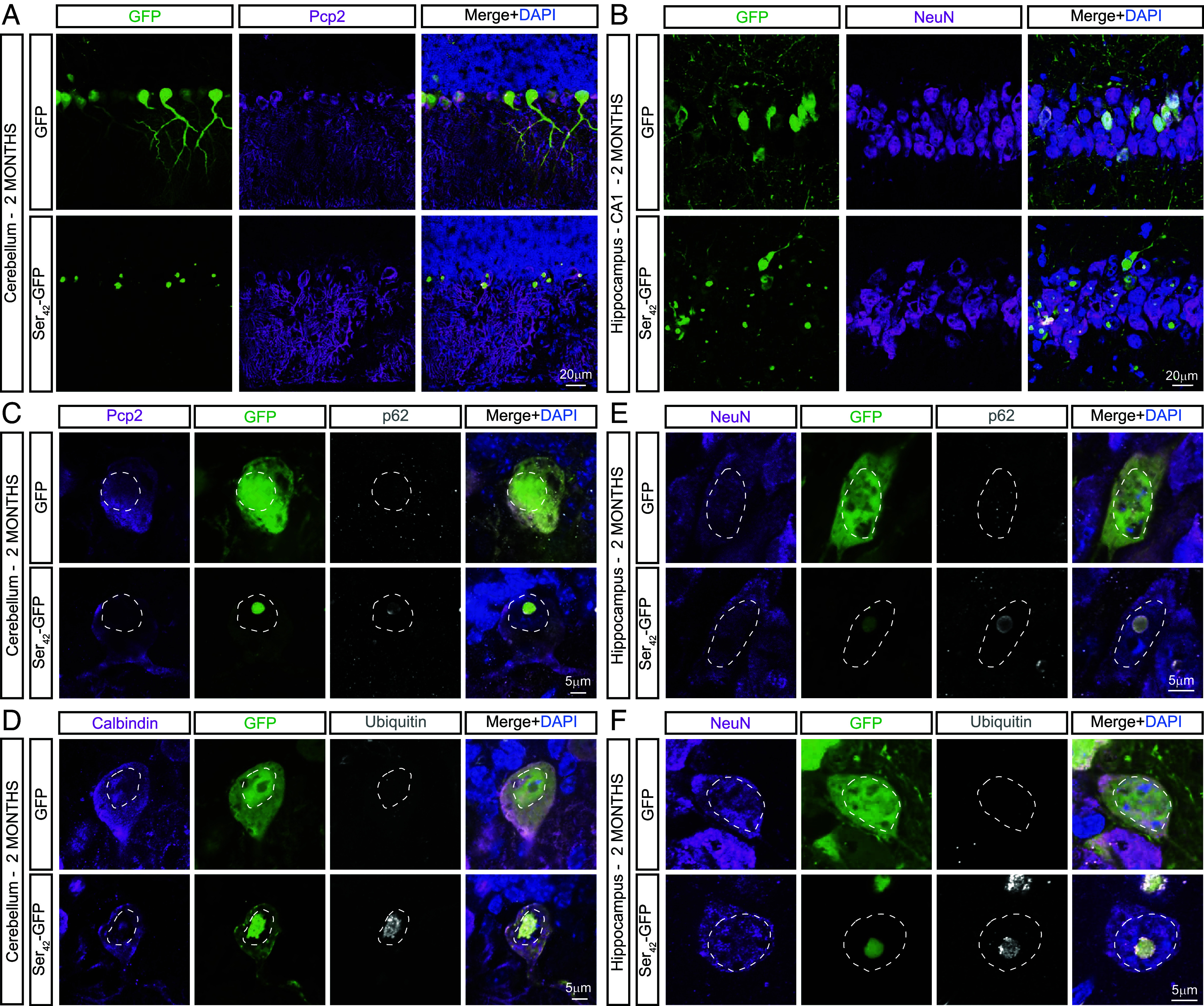
Polyserine forms assemblies in transduced neurons. (*A*) Immunostaining of DAPI (blue), GFP (green), and Pcp2 (magenta) in Purkinje cells of WT animals at 2 mo injected with 2 × 10^11^ vgs of AAV9-GFP or AAV9-Ser_42_-GFP per animal. (*B*) Immunostaining of DAPI (blue), GFP (green), and NeuN (magenta) in CA1 hippocampal neurons of WT animal at 2 mo injected with 2 × 10^11^ vgs of AAV9-GFP or AAV9-Ser_42_-GFP per animal. (*C*) Immunostaining of DAPI (blue), GFP (green), Pcp2 (magenta), and p62 (gray) in the cerebellum of WT animal at 2 mo injected with 2 × 10^11^ vgs of AAV9-GFP or AAV9-Ser_42_-GFP. The dashed outline represents the nucleus. (*D*) Immunostaining of DAPI (blue), GFP (green), calbindin (magenta), and ubiquitin (gray) in the cerebellum of WT animal at 2 mo injected with 2 × 10^11^ vgs of AAV9-GFP or AAV9-Ser_42_-GFP. The dashed outline represents the nucleus. (*E*) Immunostaining of DAPI (blue), GFP (green), NeuN (magenta), and p62 (gray) in the hippocampus of WT animal at 2 mo injected with 2 × 10^11^ vgs of AAV9-GFP or AAV9-Ser_42_-GFP. The dashed outline represents the nucleus. (*F*) Immunostaining of DAPI (blue), GFP (green), NeuN (magenta), and ubiquitin (gray) in the hippocampus of WT animal at 2 mo injected with 2 × 10^11^ vgs of AAV9-GFP or AAV9-Ser_42_-GFP. The dashed outline represents the nucleus.

To determine the localization of Ser_42_-GFP in neurons in vivo, we performed immunostaining of brain sections from AAV9-injected mice at two months of age. We observed that Ser_42_-GFP predominantly accumulated within assemblies in highly expressing Purkinje cells in the cerebellum and CA1 and DG hippocampal neurons while GFP alone maintained diffuse localization throughout transduced cells in both WT and PS19 animals (Fig. 4 *A* and *B* and *SI Appendix*, Fig. S4 *A*-*D*). Notably, while Ser_42_-GFP expression can still be detected in the cytoplasm of these neurons (Fig. 4 and *SI Appendix*, Fig. S4), large assemblies of polySer are present within the nuclei of Purkinje cells (marked by Pcp2 or calbindin positivity) and hippocampal neurons (marked by NeuN positivity) (Fig. 4 *C*–*F*). Interestingly, these intranuclear polyserine assemblies are also positive for both p62 and ubiquitin (Fig. 4 *C*–*F*) and reminiscent of the nuclear inclusions of aggregated proteins seen in polyglutamine disorders (15–18).

Thus, when overexpressed in neurons in vivo the presence of a polySer domain leads to accumulation in ubiquitin-positive nuclear inclusions which may contribute to observed toxicities.

### Polyserine Exacerbates Tau Pathology in PS19 Mice.

**Fig. 5.**
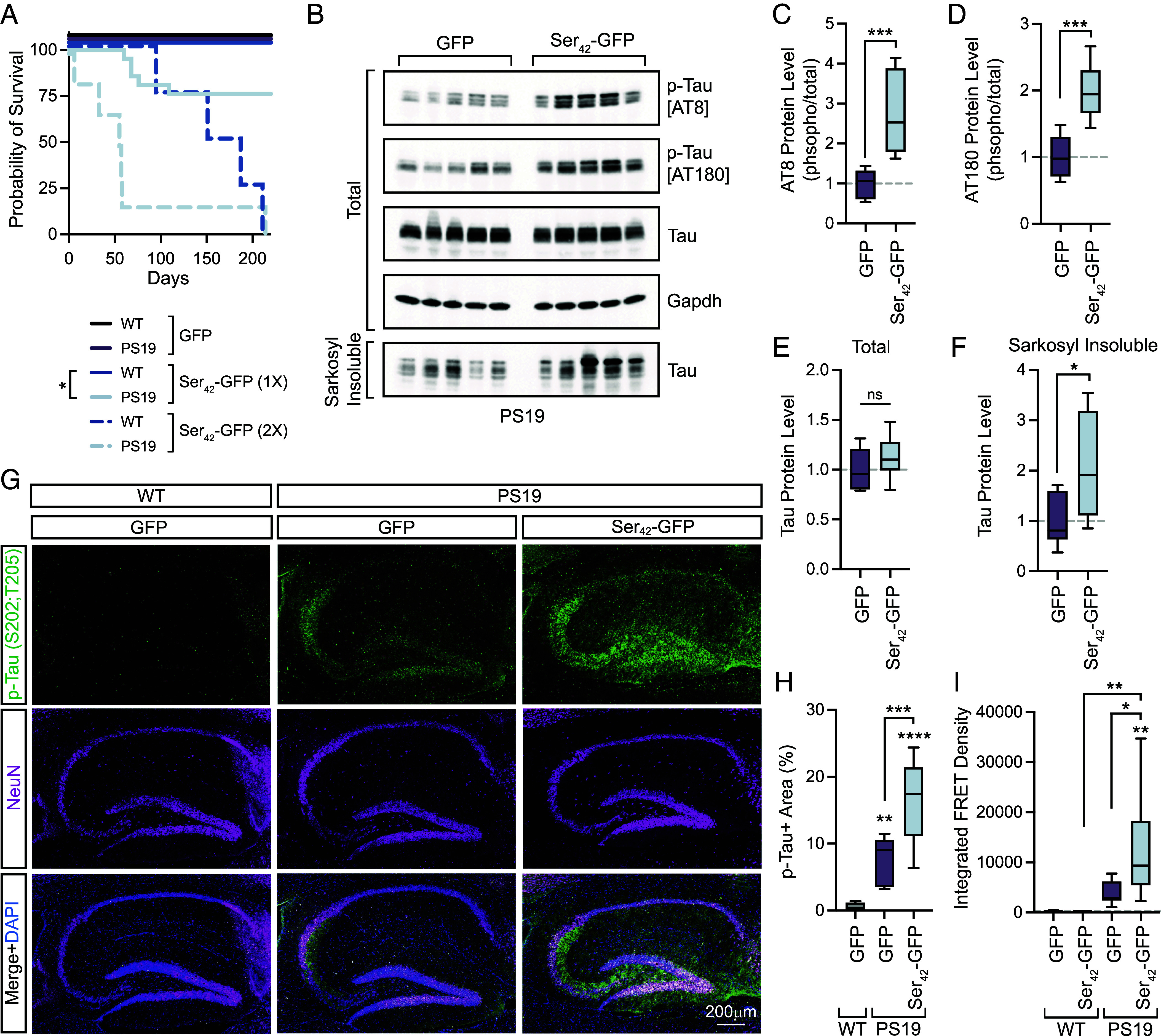
AAV9-mediated overexpression of polyserine exacerbates tau pathology. (*A*) Kaplan–Meier plot of the survival of WT and PS19 tau transgenic animals injected with GFP or Ser_42_-GFP at 1 × 10^11^ vgs (1X) [GFP: WT n = 18 (8F, 10M), PS19 n = 17 (10F, 7M), Ser_42_-GFP: WT n = 14 (8F, 6 M), PS19 n = 21 (10F, 11M)] or 2 × 10^11^ vgs (2X) [Ser_42_-GFP: WT n = 6 (2F, 4M), PS19 n = 6 (2F, 3M)] per animal at P1. Statistics performed with the Mantel–Cox test. (*B*) Western blot of phosphorylated tau (p-tau) (AT8), p-tau (AT180), total tau and GAPDH in total or sarkosyl insoluble extracts from PS19 animals injected with 1 × 10^11^ vgs of AAV9-GFP [n = 5 (3F, 2M)] or AAV9-Ser_42_-GFP [n = 5 (3F, 2M)]. (*C*) Quantification of AT8 p-tau protein levels normalized to total tau as in (*B*). The box and whisker plot represents median, interquartile range, and error determined by Tukey’s method. n = 8 animals per group. Statistics performed with the unpaired *t* test. (*D*) Quantification of AT180 p-tau protein levels normalized to total tau as in (*B*). The box and whisker plot represents median, interquartile range, and error determined by Tukey’s method. n = 8 animals per group. Statistics performed with the unpaired *t* test. (*E*) Quantification of total tau protein levels normalized to GAPDH as in (*B*). The box and whisker plot represents median, interquartile range, and error determined by Tukey’s method. n = 8 animals per group. Statistics performed with the unpaired *t* test. (*F*) Quantification of total tau levels in sarkosyl insoluble fractions normalized to GFP-injected control as (*B*). The box and whisker plot represents median, interquartile range, and error determined by Tukey’s method. n = 8 animals per group. Statistics performed with the unpaired *t* test. (*G*) Immunostaining of DAPI (blue), p-tau (AH36) (green), and NeuN (magenta) in the hippocampus of WT or PS19 at 6 mo injected with 1 × 10^11^ vgs of AAV9-GFP or AAV9-Ser_42_-GFP. (*H*) Quantification of the percent area of the hippocampus positive for p-tau (AH36) staining as in (*G*). The box and whisker plot represents median, interquartile range, and error determined by Tukey’s method. n = 8 animals per group as an average of n = 3 sections per animal [WT+GFP (3F, 5 M); PS19+GFP (4F, 4 M); PS19+ Ser_42_-GFP (5F, 3 M)]. Statistics performed with one-way ANOVA and Tukey’s multiple comparisons test. (*I*) Seeding capacity of sarkosyl insoluble fractions from brain extracts of WT and PS19 mice at 6 mo of age injected with GFP [WT n = 7 (4F, 3 M), PS19 n = 9 (4F, 5 M)] or Ser_42_-GFP [WT n = 6 (3F, 3 M), PS19 n = 7 (4F, 3 M)]. The box and whisker plot represents median, interquartile range, and error determined by Tukey’s method. Statistics performed with one-way ANOVA and Tukey’s multiple comparisons test.

We next examined whether Ser_42_-GFP altered the progression of tau pathology in PS19 mice. Notably, delivery of 1 × 10^11^ viral genomes (vgs) (1X) of Ser_42_-GFP led to early fatalities, resulting in a significant reduction in the survival of PS19 mice relative to WT controls (Fig. 5*A*). Delivery of a higher dose of 2 × 10^11^ vgs (2X) of Ser_42_-GFP then showed toxicity in both WT and PS19 animals, with PS19 animals having a shorter median survival (Fig. 3*A*). These findings demonstrate that the expression of Ser_42_-GFP reduces survival more severely in mice expressing a pathogenic human tau transgene.

Next, we monitored direct readouts of tau pathology by Western blot (Fig. 5*B* and *SI Appendix*, Fig. S5*A*). We observed that brain extracts from Ser_42_-GFP-injected PS19 animals at 6 mo of age had increased levels of phosphorylated tau (p-tau) (monitored by AT8 and AT180 antibodies) relative to GFP controls (Fig. 5 *C* and *D*). Furthermore, while there were no significant changes in total tau levels across groups, Ser_42_-GFP-expressing mice had increased sarkosyl insoluble tau (Fig. 5 *E* and *F*). Next, we performed immunostaining to measure p-tau levels specifically in the hippocampus with an antibody for Ser202/Thr205 p-tau (AH36) (Fig. 5*G*). As expected, we observed an increase in the p-tau-positive area of the hippocampus in GFP-injected PS19 mice compared to WT (Fig. 5*H*). Further, consistent with Western blotting results, we also observed a significant increase in p-tau staining in Ser_42_-GFP PS19 mice as compared to GFP (Fig. 5*H*).

We also assessed the burden of tau pathology by measuring seeding activity. To do so, we transfected total and sarkosyl fractionated brain extracts from AAV9-injected animals into HEK293 tau biosensor cells that express the tau repeat domains with a P301S mutation fused to a FRET pair (CFP/YFP), which enables quantification of tau aggregation as a measure of seeding capacity by flow cytometry (19). Importantly, we observed a baseline level of FRET signal in WT animals as well as in sarkosyl soluble fractions from WT or PS19 animals, validating our fractionation and assay (*SI Appendix*, Fig. S5*B*). We observed a significant increase in the seeding capacity of sarkosyl insoluble extracts from Ser_42_-GFP-expressing tau transgenic animals quantified by the integrated FRET density (Fig. 5*I*) or the percent of FRET-positive cells (*SI Appendix*, Fig. S5*C*).

Collectively, these results demonstrate that Ser_42_-GFP can exacerbate measures of tau pathology in a transgenic mouse model.

### Tau Aggregation Is Specifically Promoted By Polyserine.

**Fig. 6.**
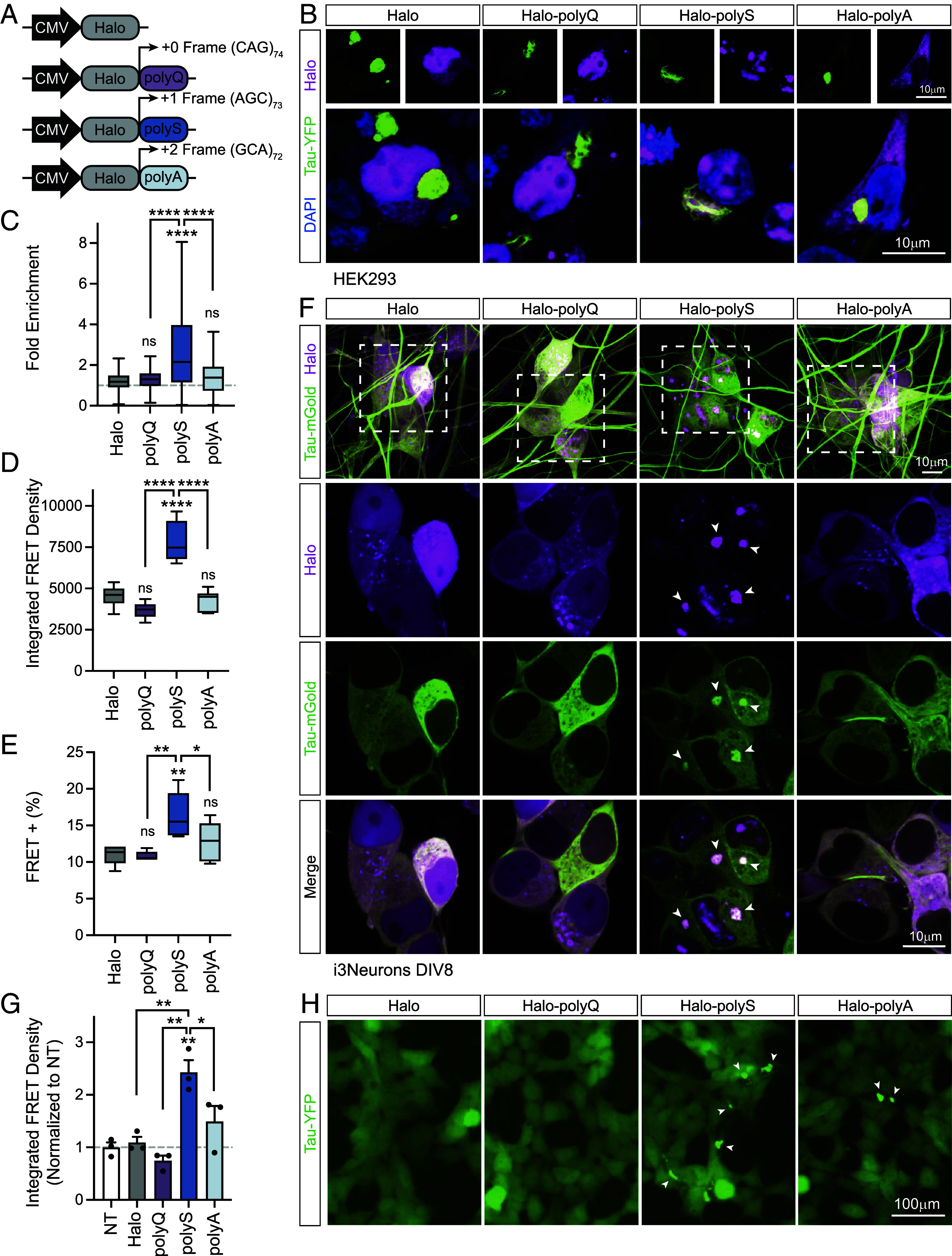
Specificity of polyserine effects on tau aggregation. (*A*) Constructs to express Halo-tagged polyglutamine (polyQ) (74 repeats), polyserine (polyS) (73 repeats), and polyalanine (polyA) (72 repeats) from different frames of a CAG expansion. (*B*) Immunofluorescence of HEK293T tau biosensor cells transfected with constructs in (*A*) labeled with JF646 Halo ligand and seeded to form tau aggregates with brain homogenate from tau transgenic mice. (*C*) Quantification of the fold enrichment of Halo-labeled proteins in cytoplasmic tau aggregates relative to the remainder of the cytoplasm as in (*B*). (Halo n = 400 cells; polyQ n = 239 cells; polyS n = 333 cells; polyA n = 196 cells). (*D*) Percentage of FRET-positive tau biosensor cells determined by flow cytometry following transfection of constructs in (*A*) and clarified brain homogenate from tau transgenic mice to induce aggregation with filtering for Halo-expressing cells. The box and whisker plot represents median, interquartile range, and error determined by Tukey’s method. n = 6. Statistics performed with one-way ANOVA and Tukey’s multiple comparisons test. (*E*) Integrated FRET density determined by flow cytometry of tau biosensor cells following transfection of constructs in (A) and clarified brain homogenate from tau transgenic mice to induce aggregation with filtering for Halo-expressing cells. The box and whisker plot represents median, interquartile range, and error determined by Tukey’s method. n = 6. Statistics performed with one-way ANOVA and Tukey’s multiple comparisons test. (*F*) Live imaging of tau-mGold and Halo-tagged proteins in human iPSC-derived neurons differentiated by doxycycline-inducible neurogenein-2 expression transduced with lentivirus-expressing human tau with P301S and S320F mutations as well as a Halo control or Halo-tagged polyA, polyQ, and polyS. The top row represents a maximum projection of a z-stack, and the remaining panels show a single z-plane of the boxed region. Arrowheads point to polyS assemblies which show enrichment of tau. (*G*) Integrated FRET density of HEK293 tau biosensor cells lipofected with cell extracts from DIV 12 iPSC-derived neurons transduced with lentiviruses as in (*F*) and normalized to a nontransduced (NT) control. Representative images shown in (*H*). Data represent mean, SEM, and individual replicates. Statistics performed with one-way ANOVA and Tukey’s multiple comparisons test. n = 3. (*H*) Live imaging of tau-YFP in HEK293 tau biosensor cells lipofected with cell extracts from iPSC-derived neurons as in (*F*). Arrowheads point to cells with tau aggregates.

To investigate whether the effect of polyserine on promoting tau aggregation is unique or shared with other repetitive proteins, we generated constructs with a Halo tag upstream of an excerpt of Huntingtin exon 1 containing a CAG repeat expansion with frameshifts thus expressing Halo tagged repeats of 74 glutamine (polyQ), 73 serine (polyS) or 72 alanine (polyA) residues (Fig. 6*A*). We transfected these constructs into HEK293 tau biosensor cells and seeded with clarified tau brain homogenate to induce tau aggregation in the presence of these repeat proteins. First, we performed imaging to determine whether polyQ, polyS, or polyA associated with cytoplasmic tau aggregates (Fig. 6*B*). The polyS frame encodes an arginine-rich stretch following the CAG expansion which may underlie the predominant localization of polyS to the nucleus. Despite this predominant nuclear localization, polyS was selectively enriched in cytoplasmic tau aggregates relative to polyQ, polyA or a Halo control (Fig. 6*C*). Next, we determined whether expression of CAG repeat expansion translation products affected the extent of tau aggregation through using flow cytometry as a readout and filtering for Halo-positive cells. Here, we observed a specific increase in the percentage of FRET-positive cells and the integrated FRET density in cells expressing polyS, but not polyQ or polyA (Fig. 6 *D* and *E*). These results indicate that polyS specifically promotes tau aggregation.

To investigate whether polySer and tau specifically associate in human neurons, we generated lentiviruses expressing Halo-tagged polyQ, polyS, and polyA as well as mGold-tagged 0N4R human tau with two disease-linked mutations P301S and S320F under control of the neuronal-specific human synapsin 1 (hSyn1) promoter. We then expressed these constructs in neurons differentiated via dox-inducible Ngn2 expression as previously described (20). Lentiviral transduction was performed 5 d following differentiation (DIV 5) and live neurons were imaged at DIV 8. We observed that polyS localized to the nucleus as well as in cytoplasmic assemblies, similar to in HEK293 cells (Fig. 6*F*). In contrast, Halo, polyQ, and polyA were observed both diffusely and in some small assemblies (Fig. 6*F*). Notably, cytoplasmic polyS assemblies enrich tau protein—a feature not observed in control or other repeat protein conditions (Fig. 6*F*). This is consistent with the direct recruitment of tau to polyserine assemblies observed in vitro (21).

The recruitment of tau to polyserine assemblies in vitro correlates with increased fibrillization (21). To determine whether the association of tau with polyserine assemblies in neurons had a similar effect, we assessed whether these cells produced seeding-competent tau fibers by lipofection of cell extracts from neuronal cultures at DIV12 into HEK293 biosensor cells. Strikingly, we observed cell extracts from Halo-polyS-expressing neurons consistently displayed seeding activity as measured through flow cytometry (Fig. 6*G*). Importantly, while polyA-expressing cell extracts also induced some aggregates, seeding activity was significantly lower than polyS (Fig. 6 *G* and *H*). Further, cell extracts from neurons not infected with lentivirus (NT), or those that express Halo or Halo-polyQ did not show measurable aggregates or seeding activity (Fig. 6 *G* and *H*). These observations suggest polyserine assemblies stimulate the formation of seeding-competent tau species in human neurons.

Collectively these findings underline that polySer is uniquely effective at promoting tau aggregation, distinct from other homopolymeric repeats of similar length expressed from CAG repeat expansions.

## Discussion

This study characterizes the toxicities of excess polySer in the mammalian brain. We observe that AAV9-mediated expression of polySer leads to reduced weight, locomotor defects, and learning deficits in WT mice (Fig. 1). Moreover, this is coincident with a loss of Purkinje cells which likely contributes to behavioral defects (Fig. 2) as well as gliosis (Fig. 3). Interestingly, a previous study showed exogenous polySer peptides lead to reduced viability of cells in vitro, vacuolar degeneration, and behavioral changes following direct delivery to the ventricles of mice (22). Collectively, these findings underline a toxic role for polySer in vivo; however, further investigation is required to elucidate the precise molecular mechanisms (*SI Appendix*, Fig. S6).

We observe that high levels of polySer lead to accumulation in neuronal nuclei (Fig. 4). These nuclear assemblies may reflect the propensity of polySer to form self-assemblies which we have previously reported in vitro and in cellular models (7, 21). Other groups have also observed that polySer forms aggregates in vitro, puncta in C. elegans and assemblies in the brains of SCA8 and HD patients (8, 9, 22–24). In this study, we observe nuclear polyserine assemblies are both p62 and ubiquitin positive, suggesting they consist of aggregated proteins that are unable to be cleared and this proteotoxic stress may contribute to the toxicity of polySer in neurons.

Our findings are pertinent as polySer expression has been identified in multiple disease contexts where it may contribute to pathobiology. Most notably, polySer is detectable in patient tissues from SCA8 and HD cases caused by CTG and CAG repeat expansions, respectively (8, 9). Due to bidirectional transcription and RAN translation, polySer could also be expressed in additional repeat expansion disorders caused by CTG/CAG expansions as suggested by detection of polySer RAN translation products SCA type 12 iPSC-derived cell lines (25). Accumulations of polySer in SCA8 postmortem tissues are mainly detected in white matter regions where they correlate with demyelination and axonal degeneration (8). While our viral-delivered polySer is expressed primarily in neurons, it is sufficient to induce locomotor dysfunction in mice featuring a pronounced loss of Purkinje cells. A key caveat is that the viral-mediated delivery of polySer in this study leads to high expression and additional studies will be required to assess whether endogenous levels of polySer as expressed in disease contexts are sufficient to induce toxicity. Nonetheless, our observations support a potential contribution of polySer toxicity to repeat expansion disorders.

This study also identifies that polySer can promote tau aggregation and pathology in the mammalian brain (Fig. 5). First, polySer expression significantly reduces survival in tau transgenic mice. Second, we observe polySer increases levels of phosphorylated, insoluble, and seeding-competent species of tau in the brain. Together, these observations demonstrate that polySer levels can impact tau pathology in mice, consistent with earlier work showing the levels of polySer expression correlate with the extent of tau aggregation in cell lines (7). We have previously shown that polySer is sufficient to increase rates of tau fibrillization in vitro and polySer or endogenous polySer containing proteins form assemblies that are preferred sites for tau aggregation in cellular models (7, 21). Importantly, endogenous polySer containing proteins such as SRRM2 and PNN colocalize with tau aggregates in cell models, animal models and postmortem samples (6, 7). Additionally, increased cytoplasmic levels of SRRM2 have been linked to inflammation and Aβ pathology (26). One possibility is that increased cytoplasmic mislocalization of polySer domain-containing proteins may accelerate the development of tau pathology; however, further studies will be required to address whether levels of these endogenous polyserine-containing proteins would be sufficient to contribute to disease progression in vivo.

While, we cannot exclude the possibility that polySer may affect tau pathology through indirect mechanisms such as increasing neuroinflammation and/or altering protein homeostasis pathways, our results suggest polySer has specific activity toward tau. Specifically, we observe polySer—but not other CAG expansion translation products—colocalizes with and exacerbates tau aggregates (Fig. 6). Further, in iPSC-derived neurons, polySer exhibits a higher propensity to form assemblies than other homopolymeric repeats and enriches tau protein increasing the production of tau seeds. Interestingly, tau pathology has been identified in some patients with polySer expression repeat expansion diseases such as HD (27) and SCA8 (28, 29) raising the intriguing possibility that polySer could also contribute to repeat expansion disease progression in a tau-dependent manner. Addressing this issue will require substantial examination of patients for both polySer expression and tau pathology, but highlights the possibility that developing our understanding of the links between polySer and tau can provide insight into disease mechanisms beyond primary tauopathies.

## Materials and Methods

### Animal Procedures.

All work with mice was performed in accordance with the NIH Guide on the Care and Use of Animals and approved by the Institutional Animal Care and Use Committee of the University of Colorado Boulder. PS19 tau transgenic mice on a C57BL/6-congenic background were obtained from Jackson mice (Strain #024841) (11) and crossed with C57BL/6 J (Strain #000664) to obtain wild-type or heterozygous experimental animals. Genotyping was performed using DNA extracted from tails with primers listed in *SI Appendix*, Table S2. As trends between groups were conserved across sexes, the aggregated data of male and female animals are presented, and specification of the number of male or female animals is reported in the figure legends.

### AAV9 Production and Delivery.

The ORFs of GFP and Ser_42_-GFP were cloned into the pAAV-CMV plasmid (Addgene #105530). Endofree plasmids were prepped with a ZymoPURE Midiprep plasmid kit (Zymo Research), and Vector BioLabs generated AAV9. AAV9 was delivered by a single injection to the right lateral ventricle at a dose of 1 × 10^11^ or 2 × 10^11^ genome copies per animal at P1 in a PBS solution containing FastGreen dye (Sigma) as previously described (30).

### Mouse Behavioral Assays.

Mice were tested on the accelerating rotarod assay as previously described (31). Briefly, animals were trained one week before their first testing at 4 mo of age, and subsequent testing was performed monthly. A 60 s warm-up was performed before 3 trials which consisted of 5 min of acceleration from 4 to 40 rotations per minute. The average of 3 trials was plotted for each animal.

Elevated plus maze assay was performed as previously described (32). Animals were moved to the testing room and singly housed with 55 dB white noise prior to testing. After 1 h, mice were placed on elevated plus maze for 5 min and monitored with EthoVision XT video tracking software. The percentage of time spent on either open or closed arms relative to the total time of testing was reported.

For open-field assay, animals were moved to the testing room and individually housed with 55 dB white noise prior to testing. Following 1 h of acclimation, animals were placed in the arena for 10 min and monitored with EthoVision XT video tracking software. The total arena size is 40 × 40cm, the small center is 30 × 30cm, and the large center is 35 × 35cm. The percentage of time spent in each portion of the arena relative to total testing time was reported.

Fear conditioning assay was performed as previously described (33, 34). Briefly, animals were acclimated for 1 h before testing, singly housed with 55 dB white noise. Animals were monitored in isolation cubicles (30” W × 17.75” D × 18.5”H) (Coulbourn) with FreezeFrame software (Actimetrics) during all testing. During training, animals were subject to two pairings of a tone (30 s, 85-dB white noise) and foot shock (2 s, 0.5 mA). The tone was (120 to 150 s and 210 to 240 s) followed by foot shock (148 to 150 s and 238 to 240 s) twice during a total session length of 5 min. House lights were on for the training period and a peppermint odor present. The following day, mice were again acclimated as described above. Cued and contextual testing were performed on the same day with a minimum of 1 h between testing for each animal. The order of cued or contextual testing was randomized. During context testing, animals were returned to identical isolation cubicles and exposed to the same conditions as the training for a total of 5 min. During cued testing, animals were placed in randomized isolation cubicles with a red light on, infrared light on, house lights off, white acrylic floor over shock grid, inserts with different display patterns over test cage walls and vanilla odor. Animals were monitored with these conditions for a total of 5 min with tone at the same times as in the training test but with no paired foot shock. The average percentage of time freezing during each 10-s training interval was reported. The average time spent freezing across the 5-min test was reported for context testing. For the baseline freezing measured before the cued stimulus, the average time spent freezing in cued testing prior to playing of the first tone (0 to 120 s) was reported. For cued testing, the average time spent freezing during both periods of tone playing was reported.

### Immunohistochemistry.

Following dissection, brains were hemisected and postfixed in 4% PFA in PBS for 24 h. Tissue was cryoprotected in 30% sucrose in PBS for 24 h before flash freezing in O.C.T. Sagittal sections were prepared on the cryostat at 30um thickness and mounted on charged slides. Staining was performed in a humidified chamber. Sections were blocked with 10% donkey serum in PBS with 0.4% Triton-X at room temperature and then incubated with primary antibodies overnight at 4 °C in blocking buffer. Following primary, slides were washed 3 × 10 min in PBS with 0.4% Triton-X and then incubated with secondary antibodies for 3 h at room temperature in blocking buffer. Following secondary, slides were washed 3 × 10 min in PBS before mounting with Fluoromount G.

TUNEL staining was performed with TUNEL Assay Kit (Fluorescence, 640 nm) (Cell Signaling) according to the manufacturer’s protocol prior to immunostaining. For DNAse treatment, sections were incubated with TURBO DNAse (1U/μL) at 37 °C for 30 min and then washed 3 × 10 min with PBS prior to TUNEL staining. TUNEL reaction was incubated for 2 h at 37 °C protected from light.

### Sarkosyl Fractionation.

Sarkosyl fractionation was performed as others previously reported (35). Briefly, flash-frozen brain tissue was homogenized with a Dounce homogenizer in 10 mM Tris-HCl (pH 7.4), 0.8 M NaCl, and 1 mM EDTA buffer supplemented with protease and phosphatase inhibitors (Roche) at a weight to volume ratio of 1:10. Lysate was centrifuged at 21,000×*g* for 10 min at 4 °C. The supernatant was incubated at room temperature with 1% sarkosyl for 1 h and then centrifuged for 1 h at 186,000×*g* at 4 °C. The pellet was washed once with PBS and then centrifuged again for 1 h at 186,000×*g* at 4 °C. The pellet was resuspended in PBS at 1/2 the starting total volume of lysate.

### Western Blotting.

Western blotting was performed as previously described.(7) Samples were boiled in 2X Laemmli sample buffer at 95 °C for 10 min and then run on precast 4 to 20% Tris-glycine gels (Invitrogen). Samples were transferred to nitrocellulose membrane using iBlot 2 gel transfer device (Invitrogen). Samples were blocked with 5% milk or 5% BSA (for phosphor blots) in TBS-T (0.1%Tween) for 1 h, incubated with primary antibodies for 2 h, washed 3 × 10 min with TBS-T, incubated with secondary antibodies in TBS-T for 1 h, and then washed 6 × 5 min with TBS-T. All steps were performed at room temperature. Blots were developed with Clarity Western ECL Substrate (Bio-Rad) and imaged with iBright imaging system (Thermo Fisher).

### DNA Constructs.

Plasmid pEGFP-Q74 was obtained from Addgene as a gift from David Rubinsztein (Addgene plasmid #40262), and a Halo tag was cloned upstream of the CAG repeats replacing GFP and inducing frameshifts to express polyglutamine, polyserine or polyalanine. Plasmid pLenti-hSyn1-mScarlet/CAAX was obtained from Addgene as a gift from Patrik Verstreken (Addgene plasmid #197592), and CAG repeat expansion products with upstream Halo tag were cloned from previous vectors downstream of hSyn1 to replace mScarlet insert.

### Cell Culture.

HEK293T tau biosensor cells were purchased from ATCC and culture in DMEM with 10% FBS, 0.2% penicillin-streptomycin at 37 °C with 5% carbon dioxide (19). Brain homogenate was transfected into cells with Lipofectamine 3000. For total and sarkosyl soluble extracts, 1 μL of sample was transfected per 24-well 24 h after plating at a density of 100 K cells/well. For sarkosyl insoluble extracts, 20 μL of the resuspended pellet was transfected per 24-well 24 h after plating at a density of 100 K cells/well. For polyserine, polyalanine, or polyglutamine experiments, 500 ng of plasmids per 24-well were transfected with Lipofectamine 3000 24 h after plating at a density of 100 K cells/well. 24 h after transfection, seeding with clarified tau brain homogenate from Tg2541 tau transgenic animals at a final concentration of 0.5 ng/μL was performed as previously described (7). TMRDirect Halo ligand (200 nM) was added at the time of seeding to label Halo-tagged proteins. For immunofluorescence imaging, HEK293T tau biosensor cells were plated on glass coverslips at a density of 125Kcells per 24 well. Plasmids (500 ng/well) and tau seeding with tau brain homogenate (1.75 ng/μL) were performed with Lipofectamine 3000, and cells were incubated for 24 h prior to fixation. At the time of transfection, JF646 Halo ligand was added (200 nM). Fixation was performed with 4% paraformaldehyde for 15 min, permeabilization in 0.5% Triton-X for 10 min, blocking in 3% BSA, 1 h labeling with DAPI in blocking buffer, 3 × 5 min washes in PBS, and then mounting with ProLong Glass.

As previously described (20), WTC-11 iPSCs expressing a doxycycline-inducible form of neurogenin-2 were cultured on Matrigel-coated plates in Essential 8 Medium supplemented with chroman (50 nM) for 24 h following thawing or passaging. Media exchange was performed daily, and passaging was performed with EDTA (0.5 mM) for expansion. For differentiation, iPSCs at 70-80% confluency were dissociated with Accutase and plated onto Matrigel coated plates (2 × 10^6^ cells per 10 cm plate) in neuronal induction media [DMEM/F12 supplemented with N2 supplement (1X), nonessential amino acids (1X), GlutaMAX (1X)] supplemented with doxycycline (2 μg/mL) and chroman (50 nM). Daily media exchanges were performed without chroman for the subsequent two days, and neuronal precursor cells were passaged with Accutase after 3 d of differentiation and frozen down in neuronal induction media with 10% DMSO for use in downstream experiments. Neuronal precursor cells were subsequently thawed and plated on glass plates coated overnight with first poly-D-lysine and then laminin in media [DMEM/F12 (50%)/BrainPhys (50%) supplemented with N21MAX (1X), GDNF (10 ng/mL), BDNF (10 ng/mL), NT-3 (10 ng/mL), laminin (1 μg/mL), doxycycline (2 μg/mL)] as previously described (36). Lentivirus was added at 5 d postdifferentiation, and half media exchange with the same formula as above was performed at 7 d postdifferentiation (DIV7). At DIV 7 JF646 Halo ligand was added to culture media (200 nM) for imaging.

### Seeding With Cell Extracts.

For seeding experiments, at DIV12 cultured neurons were washed once in PBS, then collected, and stored as cell pellets at -80C. Cells were thawed and lysed in PBS supplemented with protease inhibitor (Roche) and passed through a 25G needle. Cell pellets from neurons plated at a density of 31,000 cells at DIV 4 were lysed in 100 μL PBS, and 50 μL was sonicated in a cup horn sonicator for 5 min at amplitude 50, then transfected with Lipofectamine 3000 into HEK293 biosensor cells plated one day prior at a density of 100 K cells/24-well, and imaged and analyzed by flow cytometry 24-hours posttransfection.

### Flow Cytometry.

Flow cytometry was performed as previously reported (7). In brief, cells were trypsinized, washed with PBS, and passed through 40 μm sterile filters. Then, flow cytometry was done with a BD FACSCelesta™ Cell Analyzer using filter sets 405-450 (CFP) and 405-525 (FRET). Analysis was performed for FlowJo, and control samples were set to a false FRET percentage of 1 as previously reported (7). Reported integrated FRET density was calculated as the product of the percentage of FRET-positive cells and median fluorescence intensity.

For flow cytometry analysis of polyserine, polyalanine, or polyglutamine constructs, samples were prepared for flow cytometry as described above with the addition of the filter set 561-585 for Halo expression. Gating for the top 10% of Halo-expressing cells was performed following gating for single cells, and prior to gating for FRET positivity.

### Lentiviral Generation.

Lentivirus was generated utilizing HEK293T cells cultured as described above which were transfected one day after plating (5 × 10^6^ cells/10 cm plate) with cloned transfer plasmids (5.4 μg), pMDL6 gag/pol (3.2 μg), pRSV Rev (1.8 μg), and pVSV-G (pMD2.G) (1.8 μg) with Lipofectamine 3000. The following day, medium was removed and replaced with DMEM supplemented with 10% FBS without antibiotics. 48 h after transfection and 30 h after media change, supernatant was collected and centrifuged at 300×*g*, and the supernatant was then passed through a 45 μm filter. 1 volume of Lenti-X concentrator was added for 3 volumes of clarified supernatant and mixed by gentle inversion and then incubated overnight at 4 °C. Samples were then centrifuged at 1,500×*g* for 45 min at 4 °C, and the pellet from 1 10 cm plate of HEK293T cells was resuspended in 500 μL PBS, aliquoted, and stored at −80C. 10 μL of each concentrated lentivirus was added to well plated with 31 K neurons one day prior in a final media volume of 300 μL.

### Confocal Imaging and Image Quantification.

Imaging of immunostaining was performed on a Nikon spinning disk confocal microscope with a 10X (NA 0.45) objective and 3 μm steps using stitching and overlap to obtain composite images of the entire cerebellum or hippocampal cross-section. Images of polySer assemblies were acquired with a 40X (NA 1.15) objective. Quantification of Purkinje cell density was performed from maximum projections of scans. The number of Purkinje cells was divided by the length quantified in each lobule region or averaged across the entire cerebellum. An average density of three (four months) or two (six month) sections for each animal was calculated and reported for each animal. Quantification of the cross-sectional area of the cerebellum was performed with manual ROI selection and NIS-Elements AR (Nikon) software. Quantification of Iba1-positive area of the cerebellum or p-tau-positive area of the hippocampus was performed with a CellProfiler pipeline. Specifically, maximum projections of scans were manually annotated and masked for the cerebellum based on Pcp2 staining or the hippocampus based on NeuN staining, and then, Iba1 or p-tau positivity was defined by intensity thresholding. The percentage positive area was determined and calculated relative to the total cerebellum or hippocampal area of each section. The average value for each replicate animal was calculated from three sections per animal. Quantification of GFAP intensity was performed with a CellProfiler pipeline. Specifically, maximum projections of scans were manually annotated and masked for the cerebellum based on Pcp2 staining and quantified for the mean intensity of GFAP within that region. Imaging of immunofluorescence of HEK tau biosensor cells was performed on a Nikon spinning disk confocal microscope with a 100X (NA 1.45) objective. Quantification of the fold enrichment of Halo-labeled proteins in tau aggregates was performed using a CellProfiler pipeline. First, nuclei and cytoplasm of individual cells were segmented. Next, cells were filtered for those that express Halo-labeled protein by intensity thresholding. Tau aggregates were identified and segmented based on fluorescence intensity. The fold enrichment was calculated as the mean intensity of Halo signal within cytoplasmic tau aggregates divided by the mean Halo intensity of the remainder of the cytoplasm. For live imaging experiments, cells were maintained in a humidified chamber at 37 °C with 5% carbon dioxide, and imaging was performed on a Nikon spinning disk confocal microscope with a 100X (NA 1.45) objective with a step size of 0.5 μm and z-stack of 9 μm.

### Statistical Analysis.

Statistical tests and replicates are reported in figure legends. In figures, statistical significance is reported as follows *P < 0.05, **P < 0.01, ***P < 0.001, and ****P < 0.0001 for the defined *P*-values.

## Supplementary Material

Appendix 01 (PDF)

Movie S1. **Gait abnormality in polySer treated animals**. Gait abnormalities of an AAV9-Ser_42_-GFP treated animal in the right side of the cage at the start of the video can be observed relative to a control animal at 6 months of age.

Movie S2. **Head tilt in polySer treated animal**. Head tilt phenotype in AAV9-Ser_42_-GFP treated animal at 6 months of age.

Movie S3. **Circling phenotype in polySer treated animals**. Circling phenotype in AAV9-Ser_42_-GFP treated animals at 6 months of age.

## Data Availability

Data needed to evaluate the conclusions in the paper are included in the article and/or the supporting information.
