# Supplementary material for: Polyserine domains are toxic and exacerbate tau pathology in mice
Source: Proc Natl Acad Sci U S A. 2026 Jan 2;123(1):e2527425122. doi: 10.1073/pnas.2527425122 (PMC12773705; doi:10.1073/pnas.2527425122)
Supplement: Supplementary file 1 — Appendix 01 (PDF) [file pnas.2527425122.sapp.pdf]

PNAS

Supporting Information for
Polyserine peptides are toxic and exacerbate tau pathology in mice

Meaghan Van Alstyne[a,b], Vanessa L. Nguyen[a], Charles A. Hoeffer[c] and Roy Parker [a,b,e*]

[a]Department of Biochemistry, University of Colorado Boulder, CO, USA

[b]Howard Hughes Medical Institute, University of Colorado, Boulder, CO, USA

[c]Department of Integrative Physiology, University of Colorado Boulder, CO, USA

[e]BioFrontiers Institute, University of Colorado Boulder, CO, USA

*Corresponding author: Roy Parker

Email: Roy.Parker@Colorado.edu

This PDF file includes:

Figures S1 to S6
Tables S1 to S2
Legends for Movies S1 to S3

Other supporting materials for this manuscript include the following:

Movies S1 to S3

Figures

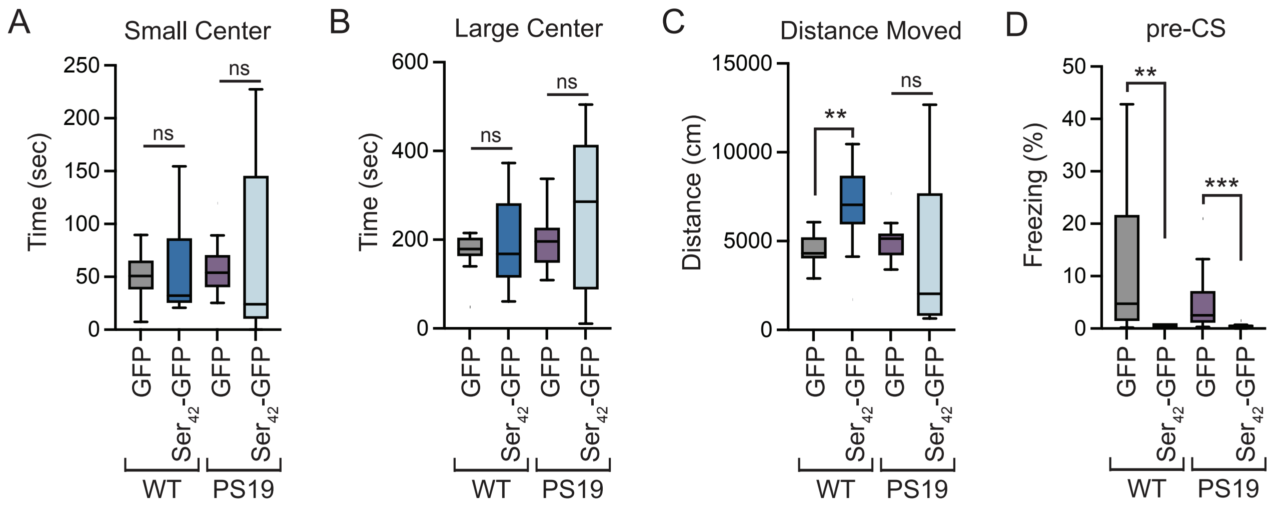

Figure S1. AAV9-mediated overexpression of polyserine leads to motor dysfunction in wild-type and tau transgenic mice. (**A**) Time spent in the small center during open-field assay at 6 months for WT (GFP n=14 (6F, 8M); Ser_{42}-GFP n=14 (7F, 7M)) and PS19 (GFP n=14 (6F, 8M); Ser_{42}-GFP n=13 (8F, 5M)) animals injected with AAV9-GFP or AAV9-Ser_{42}-GFP. Box and whisker plot represents median, interquartile range and error determined by Tukey's method. Statistics performed with Mann-Whitney test. (**B**) Time spent in the large center during open-field assay at 6 months for WT (GFP n=14 (6F, 8M); Ser_{42}-GFP n=14 (7F, 7M)) and PS19 (GFP n=14 (6F, 8M); Ser_{42}-GFP n=13 (8F, 5M)) animals injected with AAV9 as in (A). Box and whisker plot represents the median, interquartile range and error determined by Tukey's method. Statistics performed with Mann-Whitney test. (**C**) Distance moved in open-field assay at 6 months for WT (GFP n=14 (6F, 8M); Ser_{42}-GFP n=14 (7F, 7M)) and PS19 (GFP n=14 (6F, 8M); Ser_{42}-GFP n=13 (8F, 5M)) animals injected with AAV9 as in (A). Box and whisker plot represents median, interquartile range and error determined by Tukey's method. Statistics performed with Mann-Whitney test. (**D**) Percent freezing during period prior to cued stimulus (pre-CS) following conditioning in fear conditioning assay for WT (GFP n=11 (3F, 8M); Ser_{42}-GFP n=7 (4F, 3M)) and PS19 (GFP n=14 (5F, 9M); Ser_{42}-GFP n=8 (5F, 3M)) animals injected with AAV9-GFP or AAV9-Ser_{42}-GFP. Box and whisker plot represents median, interquartile range and error determined by Tukey's method. Statistics performed with Mann-Whitney test.

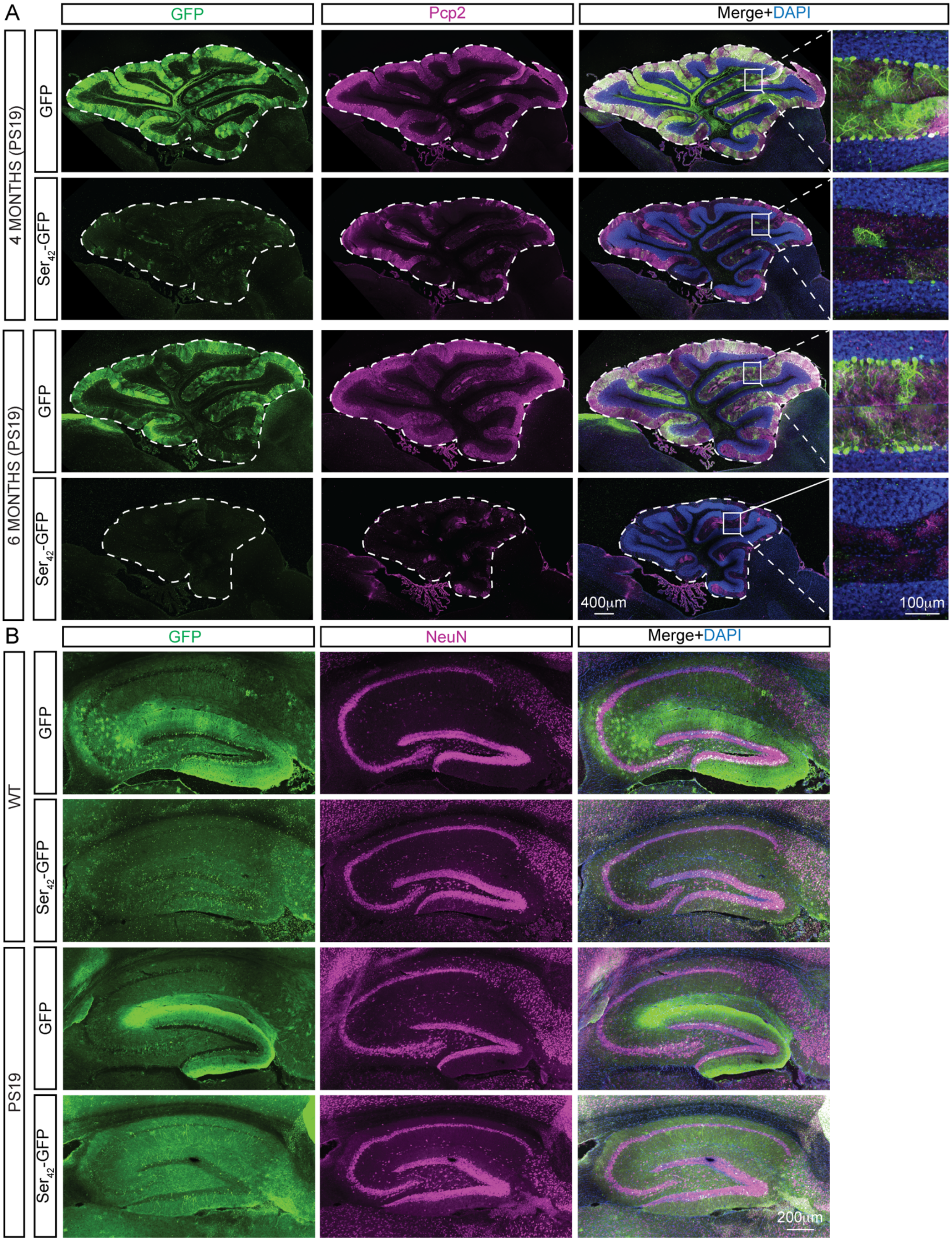
A
GFP
Pcp2
Merge+DAPI
4 MONTHS (PS19)
GFP
Ser42-GFP
6 MONTHS (PS19)
GFP
Ser42-GFP
400µm
100µm
B
GFP
NeuN
Merge+DAPI
WT
GFP
Ser42-GFP
PS19
GFP
Ser42-GFP
200µm

Figure S2. Polyserine expression induces Purkinje cell loss. (**A**) Immunostaining of DAPI (blue), GFP (green) and Pcp2 (magenta) in the cerebellum of PS19 animals at 4 and 6 months of age injected with $1x10^{11}$ vgs of AAV9-GFP or AAV9-Ser_{42}-GFP at P1 by ICV injection. (**B**) Immunostaining of DAPI (blue), GFP (green) and NeuN (magenta) in the hippocampus of WT and PS19 animals at 6 months of age injected with $1x10^{11}$ vgs of AAV9-GFP or AAV9-Ser_{42}-GFP at P1 by ICV injection.

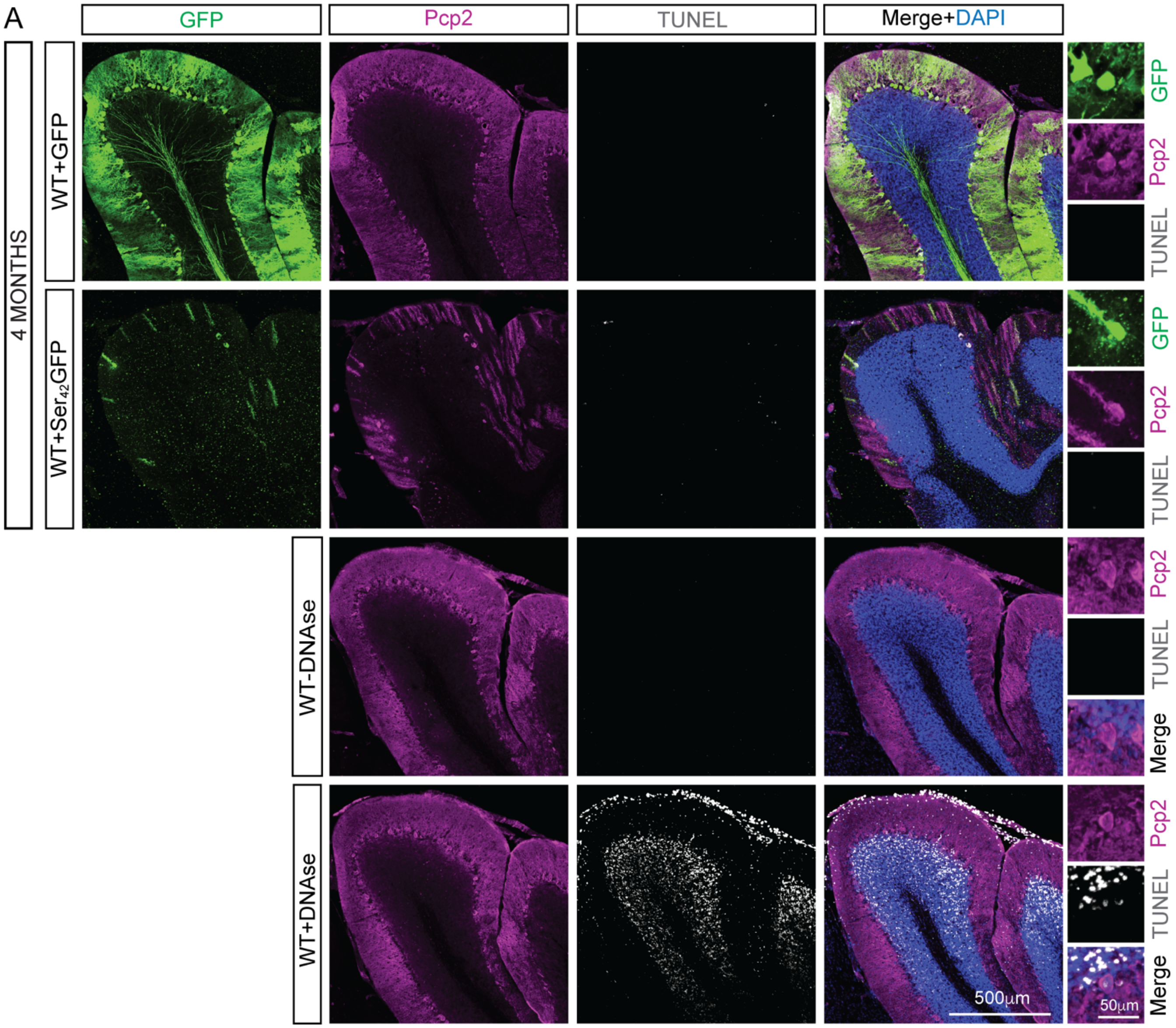

Figure S3. Polyserine expression does not lead to DNA fragmentation in Purkinje cells. (**A**) Immunostaining of DAPI (blue), GFP (green), Pcp2 (magenta) and TUNEL (grey) in the cerebellum of WT animals at 4 months of age injected with $1x10^{11}$ vgs of AAV9-GFP or AAV9-Ser_{42}-GFP at P1 by ICV injection (upper panels). Immunostaining of DAPI (blue), Pcp2 (magenta) and TUNEL (grey) in the cerebellum of WT animals at 6 months of age with or without DNAse treatment as a positive control (lower panels).

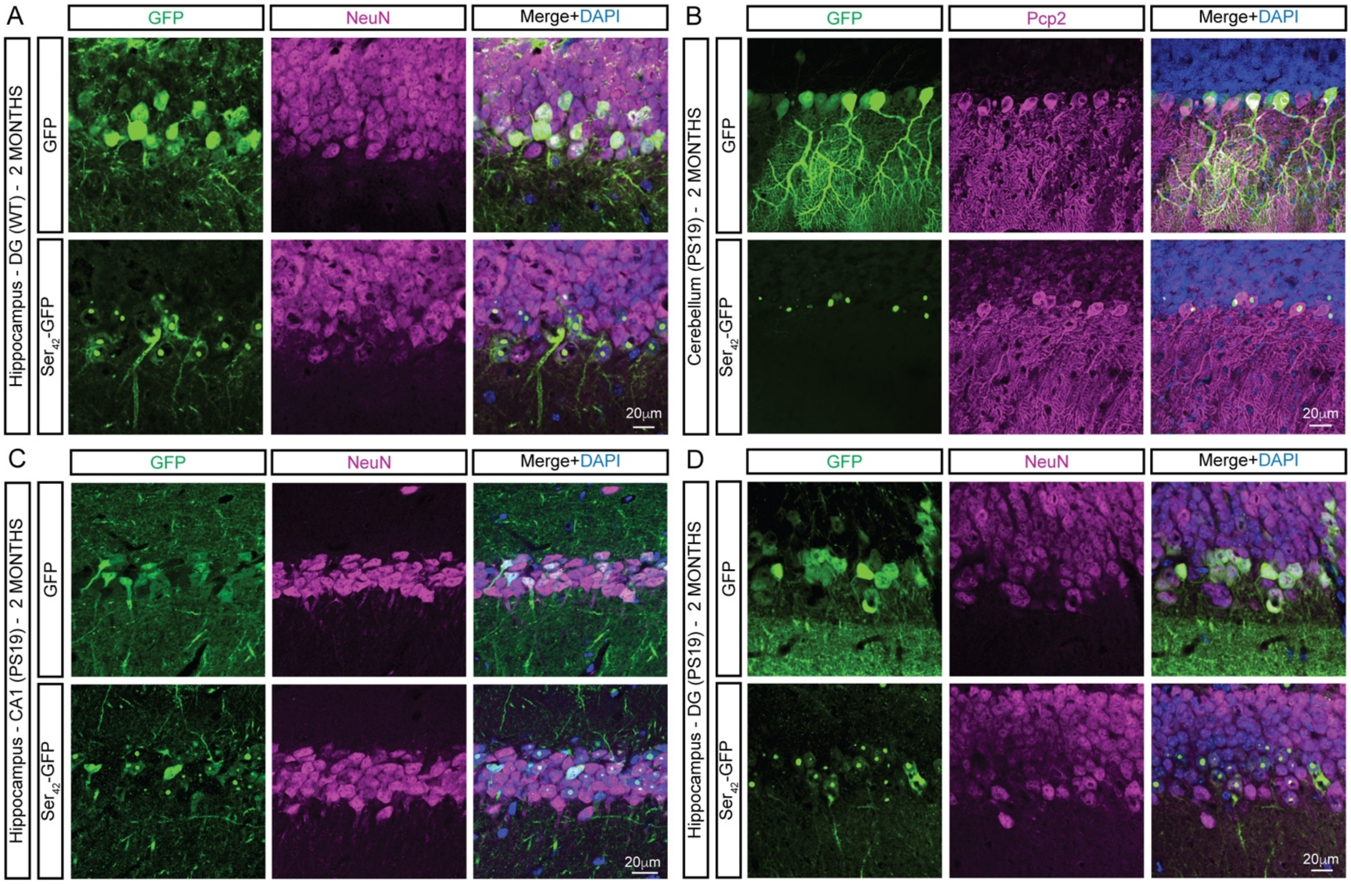

Figure S4. Polyserine forms assemblies in wild-type and tau transgenic mice. (**A**) Immunostaining of DAPI (blue), GFP (green) and NeuN (magenta) in DG hippocampal neurons of WT animals at 2 months injected with $2x10^{11}$ vgs of AAV9-GFP or AAV9-Ser_{42}-GFP per animal. (**B**) Immunostaining of DAPI (blue), GFP (green) and Pcp2 (magenta) in Purkinje cells of PS19 animals at 2 months injected with $2x10^{11}$ vgs of AAV9-GFP or AAV9-Ser_{42}-GFP per animal. (**C**) Immunostaining of DAPI (blue), GFP (green) and NeuN (magenta) in CA1 hippocampal neurons of PS19 animals at 2 months injected with $2x10^{11}$ vgs of AAV9-GFP or AAV9-Ser_{42}-GFP per animal. (**D**) Immunostaining of DAPI (blue), GFP (green) and NeuN (magenta) in DG hippocampal neurons of PS19 animals at 2 months injected with $2x10^{11}$ vgs of AAV9-GFP or AAV9-Ser_{42}-GFP per animal.

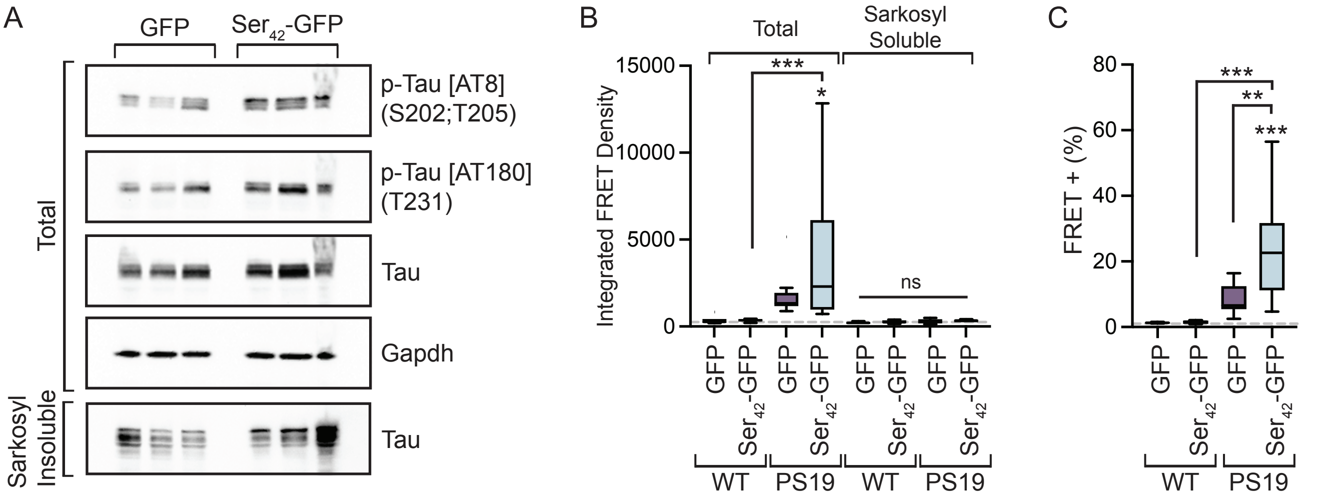

Figure S5. Polyserine forms assemblies and increases seeding capacity in tau transgenic mice. (**A**) Western blot of phosphorylated tau (p-tau) (AT8), p-tau (AT180), total tau and GAPDH in total or sarkosyl insoluble extracts from PS19 animals injected with $1x10^{11}$ vgs of AAV9-GFP (n=3 (2F, 1M)) or AAV9-Ser_{42}-GFP n=3 (2F, 1M)). Quantified in Figure 5C-F. (**B**) Integrated FRET density of HEK biosensor cells following transfection of total and sarkosyl soluble fractions from brain extracts of WT and PS19 mice at 6 months of age injected with $1x10^{11}$ vgs of AAV9-GFP (WT n=7 (4F, 3M), PS19 n=9 (4F, 5M)) or Ser_{42}-GFP (WT n=6 (3F, 3M), PS19 n=7 (4F, 3M). Data represents median, interquartile range and error determined by Tukey's method. Statistics were performed with one-way ANOVA and Tukey's multiple comparisons test to compare total or sarkosyl soluble groups. (**C**) Percentage of FRET positive cells following transfection of sarkosyl insoluble brain extracts of WT and PS19 mice at 6 months of age injected with $1x10^{11}$ vgs of AAV9-GFP (WT n=7, PS19 n=9) or AAV9-Ser_{42}-GFP (WT n=6, PS19 n=7). Box and whisker plot represents median, interquartile range and error determined by Tukey's method. Statistics were performed with one-way ANOVA and Tukey's multiple comparisons test to compare total or sarkosyl soluble groups.

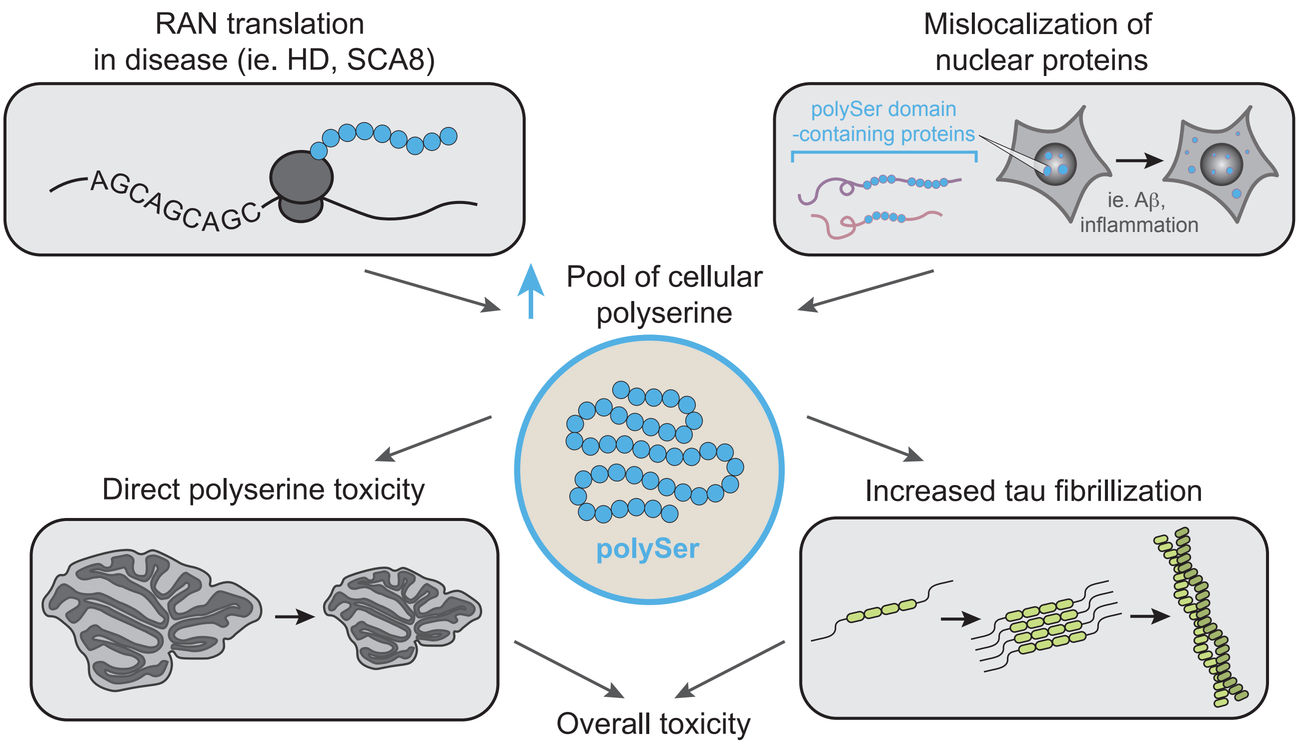

Figure S6. Model of polySer sources and toxicity outcomes. PolySer can be expressed due to RAN translation in repeat expansion diseases such as SCA8 and HD. Cytosolic levels of polySer can also increase due to polySer-domain containing nuclear proteins mislocalizing in response to stressors such as Aβ or inflammation. Increases in the pool of polySer can have direct deleterious effects such as the loss of Purkinje cells which occurs through unknown mechanisms and/or promote tau fibrillization and pathology. The additive effect of such pathways amounts to the overall toxicity induced by polySer.

Tables

Table S1. Antibodies used in the study.

Target	Manufacturer	Catalog #
DAPI	Invitrogen	62248
Mouse anti-Pcp2	Sigma	sc-137064
Chicken anti-GFP	Aves	GFP-1020
Rabbit anti-p62	MBL	PM045
Mouse anti-Ubiquitin	Cell Signaling	3936S
Rabbit anti-calbindin	Swant	CB38a
Rabbit anti-Iba1	Wako	019-19741
Mouse anti-GFAP	Agilent	Z033429-2
Guinea pig anti-NeuN	Synaptic Systems	266 004
Rabbit anti-tau (K18)	Abcam	Ab218314
Mouse anti p-tau (AT8)	Thermo Scientific	MN1020
Mouse anti p-tau (AT180)	Thermo Scientific	MN1040
Mouse anti-GAPDH	Millipore Sigma	MAB374
Rabbit anti p-tau (AH36)	Stress Marq	SMC-601D
Anti-rabbit HRP	Cell Signaling	7074S
Anti-mouse HRP	Cell Signaling	7076S
488 anti-chicken	Jackson Labs	703-545-155
CY3 anti-rabbit	Jackson Labs	711-165-152
CY3 anti-mouse	Jackson Labs	715-165-150
CY5 anti-guinea pig	Jackson Labs	706-175-148
647 anti-rabbit	Jackson Labs	111-605-045

Table S2. Genotyping primers used in the study.

Target	Common Forward (5'-3')	WT Reverse (5'-3')	Mut Reverse (5'-3')
PS19	TTGAAGTTGGGTTATCAATTTGG	TTCTTGGAACACAAACCATTTC	AAATTCCTCAGCAACTGTGGT

Movie S1 (separate file). Gait abnormality in polySer treated animals. Gait abnormalities of an AAV9-Ser$_{42}$-GFP treated animal in the right side of the cage at the start of the video can be observed relative to a control animal at 6 months of age.

Movie S2 (separate file). Head tilt in polySer treated animal. Head tilt phenotype in AAV9-Ser$_{42}$-GFP treated animal at 6 months of age.

Movie S3 (separate file). Circling phenotype in polySer treated animals. Circling phenotype in AAV9-Ser$_{42}$-GFP treated animals at 6 months of age.
